# Introduction of a Structured Reporting Protocol and Surgical Checklist for Rezum Water Vapor Therapy (VAPOR-SRP)

**DOI:** 10.3390/jcm14238431

**Published:** 2025-11-27

**Authors:** Jan Ebbing, Viktor Alargkof, Christian Engesser, Anas Elyan, Hans-Helge Seifert, Nicola Keller, Brigitta Gahl, Pawel Trotsenko, Christian Wetterauer

**Affiliations:** 1 Department of Urology, University Hospital Basel, 4031 Basel, Switzerlandchristian.wetterauer@usb.ch (C.W.); 2 Faculty of Medicine, University of Basel, 4001 Basel, Switzerland; 3 Surgical Outcome Research Center, University Hospital Basel, 4001 Basel, Switzerland; brigitta.gahl@usb.ch; 4 Department of Urology, Medical University of Graz, 8010 Graz, Austria; 5 Department of Medicine, Faculty of Medicine and Dentistry, Danube Private University, 3500 Krems, Austria

**Keywords:** benign prostatic hyperplasia, prostatic hyperplasia/surgery, checklist

## Abstract

**Background/Objectives**: Rezum water vapor therapy for benign prostatic obstruction lacks standardized documentation, complicating data comparison. This study evaluates the completeness of non-standardized Rezum operative reports and validates a novel Rezum—Structured Reporting Protocol (SRP) to enhance documentation quality. **Methods**: Following the establishment of content validity, the SRP—which includes detailed diagrams for various prostatic urethral lengths (PUL) and intravesical prostatic protrusion (IPP) to document injection sites, along with a comprehensive 10-item checklist capturing factors that may influence outcomes—was retrospectively applied to 100 Rezum cases. Operative videos and non-standardized reports were analyzed and compared against the SRP. For criterion validity, inter-rater reliability was evaluated through a blinded review of 20 cases by three Rezum users and the protocol development panel, comparing checklist item ratings. **Results**: Median number of injections was 4.0 (IQR: 2–6), injection density was 12.7 (IQR: 10–16.7) mL (PVOL)/injection, and injection interval was 0.7 (IQR: 0.5–1) cm (PUL)/injection. Variations in injection techniques were noted, including non-standard locations in 10% of cases and alternating injection sequences between lobes in 22%. Only 30% of reports detailed injection sites accurately. The intraclass coefficient for the rating of PUL was 0.94 (95% CI: 0.89–0.97). The Fleiss Kappa for MLE and IPP was 0.84 (95% CI: 0.66–1.02) and 0.85 (95% CI: 0.67–1.03), respectively. The agreement rate was 93% for bladder neck/urethra morphology and 100% for injection sequence. Kendall’s W was 0.37 (*p* = 0.343) for the item of injection sites. **Conclusions**: Variability in Rezum surgical techniques was observed, particularly in injection density, injection intervals, and precise injection locations, as well as in the structured information of non-SRP-standardized operative reports. Content validity of the SRP was achieved, leading to high inter-rater reliability in its application. The SRP promotes the standardization and completeness of Rezum data, thereby supporting improved, consistent, and high-quality Rezum documentation.

## 1. Introduction

Rezum is a novel, minimally invasive therapy for benign prostatic obstruction (BPO) using injections of water vapor in the prostate, to ablate the tissue via conduction-based transfer of thermal energy denaturing prostate cells. Its main benefits include a short procedure time, preservation of sexual function, and the possibility to perform the intervention in local anesthesia or analgosedation (often as an in-office procedure) [1,2].

Nevertheless, Rezum is included in the 2025 European Association of Urology (EAU) Guidelines as an alternative ablative technique under investigation, without receiving any recommendation. The risk factors for treatment failure remain unclear and the EAU panel calls for randomized controlled trials to evaluate the clinical utility of Rezum [3].

Physician certification to perform Rezum according to current manufacturer guidelines requires completing a training course that outlines a standardized procedural technique [4], simulator training, and completion of 10 procedures with a Boston Scientific representative with no significant complications. It is recommended to begin injections 1 cm distal to the bladder neck and reinject in 1 cm intervals, ending proximal to the verumontanum. Injections should be completed on each lobe, taking advantage of latent heat, before proceeding to the next lobe [4]. Additional injections at a 45° angle (lateral to medial) may be applied to treat a median lobe enlargement (MLE). It is noted that each injection should treat the bulk of targeted tissue, and the total number of vapor treatments can be customized based on the length of the enlarged prostatic tissue and gland configuration [4].

To date, no standardized system exists to capture or document Rezum operative technique, such as the number, spacing, or pattern of injections, and current recommendations focus on general procedural guidance rather than structured reporting or technique quantification. The lack of a standardized method for analyzing intraoperative findings and injection patterns may hinder data interpretation in future research. Variations in the number, technique, and precise location of Rezum injections can depend on the surgeon [4] and their impact on functional outcomes and complication rates remains unclear. Detailed information on intraoperative techniques is often unavailable and challenging to extract from operative notes.

To address this, we introduce a Rezum—Structured Reporting Protocol (SRP) that includes a diagram to document injection sites and a surgical checklist. We performed content validity, investigated inter-rater reliability and applied this “Rezum-SRP” to a diverse cohort of patients treated with Rezum to evaluate variations in injection techniques and assess the SRP’s effectiveness in providing information compared to conventional operative notes.

## 2. Materials and Methods

### 2.1. Ethics

The study was approved by the local ethical commission (EKNZ, ID-2022-02116, 22 November 2022) and was conducted in accordance with the Declaration of Helsinki.

### 2.2. Study Design

A three-step approach was followed to develop and test the Rezum-Structured Reporting Protocol (VAPOR-SRP) and surgical checklist. First, content validity of the reporting protocol was established by an expert panel through an iterative consensus procedure and structured assessment of SRP-item relevance. In this phase, preliminary versions of the diagrams and checklist were repeatedly reviewed, commented on, and refined until all panel members agreed that no essential elements were missing and that all items were clearly understandable. Second, the finalized SRP was applied to operation videos, and third, it was compared with the corresponding written operative reports to evaluate its feasibility, its reliability across users, and its information yield. This validation approach was chosen because no other standardized documentation tool existed for Rezum water vapor therapy. The design therefore aimed to ensure that the SRP captured all relevant intraoperative parameters (content validity), could be interpreted consistently across users (inter-rater reliability), and provided a higher quality information yield than conventional operative notes (comparative assessment). This combined methodological framework aimed to validate the SRP as an instrument, while at the same time exploring actual variability in surgical technique within a real-world cohort.

### 2.3. Rezum—Structured Reporting Protocol: Diagram and Surgical Checklist—Content Validity

An expert panel of urologists specializing in BPO surgery, including certified high-volume Rezum practitioners and a leading Rezum expert user, employed an iterative approach to create five diagrams and a 10-item surgical checklist. The manufacturer’s criteria for Rezum Center of Excellence designation include quarterly therapy reviews with a Boston Scientific representative, submission of outcome data for at least 25 patients per year with a ≥25% mean international prostate symptom score (IPSS) improvement, and completion of a minimum of 75 Rezum procedures annually. The panel designed diagrams tailored to specific prostatic urethral length (PUL) groups (1–4 cm, 4.5–6 cm, 6.5–8 cm, 8.5–10 cm) that assist in the structured documentation of injection sites. The diagrams provide injection site options in 0.5 cm intervals, various injection planes (along the 90° horizon or dorsal/ventral to the 90° horizon for lateral lobe injections; along the midline or lateral to the midline for MLE injections), injection angles and consider safety zones according to the manufacturer’s guidelines. An additional optional diagram was created for cases involving treated intravesical prostatic protrusion (IPP), accounting for 9 equal area zones. Each diagram includes a legend, and the 10-item surgical checklist that addresses essential factors potentially influencing Rezum treatment outcomes and that should be included in a comprehensive surgical documentation of the Rezum procedure. During this process, particular attention was given to ensuring that the diagrams remained intuitive to read at a glance, and that the checklist items could be completed rapidly in routine clinical practice without substantially prolonging documentation time. The content of the diagrams and 10-item checklist was developed based on the manufacturer’s guidelines, operator certification modules, published studies, and expert recommendations. To assess content validity, each expert reviewed the relevance and comprehensiveness of the included items to ensure that the diagram and checklist covered all possibly relevant aspects of the procedure, thereby reaching a consensus for the Rezum-SRP presented here.

### 2.4. Data Collection, SRP Application, Comparison to Written Operative Reports, Analysis and Statistical Methods

All patients treated with Rezum (Boston Scientific, Marlborough, MA, USA) at a single institution from December 2020 to September 2022 were identified. Cases with missing data, unavailable operation videos, or documented refusal to participate in research were excluded. Demographic and clinical data were collected. Prostate volume (PVOL) was measured using transrectal or transabdominal ultrasound (US) and/or magnetic resonance imaging (MRI), with MRI measurements preferred in cases of discrepancies with US. Descriptive statistics were used to report data, with continuous variables shown as medians and interquartile ranges, and categorical variables as absolute numbers and percentages. Injection density, defined as the PVOL-to-injection ratio, and injection interval, defined as the PUL-to-injection ratio, were calculated per patient.

The SRP was applied to recorded Rezum operation videos, assessing checklist items (Table 1) that could be evaluated based on video material (items 2, 4, 5, 6, 7, and 8). To assess the inter-rater reliability of the SRP, 20 videos with no deviation from the standard procedure (according to checklist item 9) were reviewed by three blinded Rezum users and the expert panel that developed the protocol. The checklist items were individually rated by the three users and through consensus by the expert panel. The rating of each item was compared among all participants, including the three blinded users and the expert panel members. Metric data were analyzed using the intraclass correlation coefficient, while Fleiss’ Kappa was applied for categorical data to assess agreement among all participants. For the item of injection points (item 8), benchmark ratings established by the expert panel served as the comparison standard and were compared against the three users. To evaluate agreement on item 8, which involved ordinal data, a weighted Kendall’s W was used. High partial agreement (weight = 0.75) was assigned when injections were located on the same axis (90° horizon or longitudinal midline) but differed by a longitudinal distance between injection sites of ≥0.5 cm and <1 cm. Moderate partial agreement (weight = 0.5) was assigned when injections were on the same axis but differed by a distance of ≥1 cm and <1.5 cm. No agreement (weight = 0) was assigned if the injection axis differed or the distance was ≥1.5 cm.

In addition, the written operative reports corresponding to each case were assessed to evaluate how consistently procedural parameters were documented in routine clinical practice. For this purpose, the SRP checklist items were screened in each operative note, and the presence, absence, or qualitative description of each item was recorded. Specifically, it was evaluated whether the prostate volume and the bladder neck–verumontanum distance were documented, whether the presence or absence of median lobe enlargement (MLE) and intravesical prostatic protrusion (IPP) was mentioned, and whether injection-related parameters, including injection sequence, total number of injections per lobe, and precise injection positions (distance from the bladder neck, distance to the verumontanum, and spacing between injection sites), were described. It was also noted whether any vapor leakage was documented.

## 3. Results

**Figure 1:**
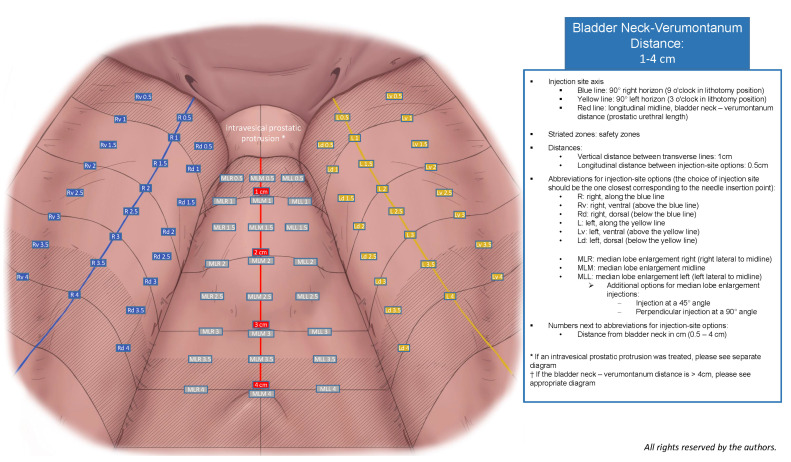
VAPOR-SRP diagram for the documentation of injection sites for PULs of 1–4 cm.

The SRP diagram documenting injection sites for PULs of 1–4 cm is shown in Figure 1, and the finalized 10-item surgical checklist appears in Table 1. Diagrams for IPP and longer PULs are included in the supplement (Supplementary Figures S1a–c and S2). These graphical tools represent the final output of the iterative content-validity process, during which the expert panel refined the layout, interval definitions, and safety margins until consensus was achieved. The panel confirmed that multiple diagrams were necessary to accommodate the substantial anatomical variability encountered across different PUL groups and to ensure that injection-site documentation remained anatomically correct and clinically interpretable, capturing all relevant injection options while maintaining a standardized visual structure. In addition, a dedicated diagram was required for cases involving the treatment of an intravesical prostatic protrusion, as the three-dimensional configuration and inward projection of IPP tissue differ substantially from those of lateral or median lobes and therefore necessitate a distinct zonal representation. Feedback from early pilot users informed minor modifications to diagram labeling, clarity of safety margins and documentation of injection sites. Table 2 summarizes patient and Rezum procedure characteristics.

**Table 1:** Surgical checklist for the Rezum procedure.

Item	Classification	Description
***Prostate measurements***		*To estimate the number of injections.*
(1) Prostate volume	Provide prostate volume in mL.	Describe the modality used to measure (e.g., TRUS, TAUS, MRI).
(2) Prostatic urethra length	Provide the bladder neck—verumontanum distance in cm.	Measure in cm with the Rezum device.
***Prostate anatomy***		*To estimate the location of injections.*
(3) Lateral lobe morphology	Describe the lateral lobe macroscopic appearance.	Identify areas with focal hypertrophy, asymmetry crossing the midline, and bulging protrusion and designate them on the diagram (Figure 1).
(4) Median lobe enlargement	Note the presence vs. absence of MLE.	If MLE is present, provide length measurement in cm with Rezum device from bladder neck to apical end.
(5) Intravesical prostatic protrusion	Note the presence vs. absence of IPP.	If IPP is present, provide height and width measurements in cm with the Rezum device according to the diagram (Figure S2).
***Bladder neck & urethra morphology***		*To assess for other LUTS etiology.*
(6) Bladder neck and urethra	Describe the appearance of the bladder neck. Describe the appearance of the urethra.	Is the bladder neck wide vs. narrow, flat vs. steep, fibrotic, or normal appearing? Is there any urethral stricture; if yes, provide the location and length of stricture, describe if passable with the device vs. requiring additional procedure, e.g., dilatation or urethrotomy.
***Surgical technique***		*To describe the precise technique and location of injections and to calculate the injection density and injection interval.*
(7) Sequence of injections	Describe the injection sequence.	Were all injections completed on one lobe before beginning injections on the contralateral lobe (sequential) or were injections performed alternating between lobes?
(8) Documentation of injections	Select the injection sites on the diagram in the order of administration (Figure 1 and Figure S2).	Describe the injection time in seconds for each injection. Describe whether vapor leakage occurred vs. not; if yes, mention if the injection was repeated and designate the injection site on the diagram.
***Deviation from standard procedure***		*To describe other intraoperative parameters that may influence the outcome of the Rezum procedure.*
(9) Additional procedure(s) and adverse event(s)	Was only standard Rezum performed vs. any additional surgical procedure. Were there any intraoperative AE(s).	State whether the additional procedure was scheduled or if performed due to incidental findings. (e.g., TUR of MLE or IPP, DVIU or stricture dilatation). State if any non-standard injections were performed (e.g., injections in the ventral prostate). Use a standardized classification, (e.g., ClassIntra®) to report intraoperative adverse events. Could the operation be completed as planned? Was abortion of surgery necessary; if yes, state the reason (e.g., intraoperative AE, unexpected findings, defect of the device). Was there adequate visibility? Was there any coagulation necessary; if yes, designate the site of most relevant bleeding (mucosal or from the injection channel).
***Sign out***		*To facilitate a safe postoperative course and to evaluate for (postoperative) factors that may influence the outcome of the Rezum procedure.*
(10) Sign out	State type of anesthesia and antibiotic prophylaxis/therapy. State the operative/procedure time. State the bladder catheter. Give postoperative instructions.	Describe the type of anesthesia methodology utilized (e.g., spinal anesthesia, general anesthesia, analgosedation or local anesthesia (intraprostatic vs. periprostatic) and, if applicable, the device used to perform local anesthesia, e.g., Schelin^®^ catheter). In instances of local anesthesia record the active substance and the quantity (volume) administered in the anesthesia protocol. Describe the type of antibiotic prophylaxis/therapy used (specify the active substance and dosage). Document the operative time, the procedure time (defined by start of first injection—end of last injection) and, if applicable, the interval between end of local anesthesia and start of first injection. State any treatments to be prescribed postoperatively (management of medications: e.g., antibiotics, BPE, anticoagulation, anti-inflammatories). State if a urinary catheter was placed, give instructions for catheter management, and note the scheduled date of catheter removal.

AE, adverse event; BPE, benign prostatic enlargement; DVIU, direct vision internal urethrotomy; IPP, intravesical prostatic protrusion; LUTS, lower urinary tract symptoms; MLE, median lobe enlargement; MRI, magnetic resonance imaging; TAUS, transabdominal ultrasound; TRUS, transrectal ultrasound; TUR, transurethral resection.

**Table 2:** Patient and Rezum procedure characteristics.

Parameter	All Patients *n* = 100
	median (range)
Age, years	69.1 (47.4–91)
Prostate volume, mL	52.0 (20–220)
Prostatic urethral length, cm	3.0 (1.5–6.5)
Total injections, *n*	4.0 (2–10)
Injection density, mL	12.7 (3.3–30)
Injection interval, cm	0.7 (0.5–1.5)
Operation time, min	13.0 (3–43)
Type of anesthesia	*n* (%)
Analgosedation	55 (55%)
General	26 (26%)
Local	19 (19%)

Within the study period, 118 patients underwent Rezum treatment, with 100 patients (85%) meeting the inclusion criteria for this study. Rezum was the first BPO intervention for all patients. Rezum treatments targeted prostates < 80 mL in 81% of cases. MLE was present in 32% of cases and IPP in 13%. MLE was treated in 87.5% (28/32; one of those received a bipolar transurethral resection) and IPP was treated in 54.0% (7/13; two of those received a bipolar transurethral resection).

The reviewed videos included nine cases with a PUL of 1–4 cm, five cases with a PUL of 4.5–6 cm, three cases involving the treatment of an MLE, and three cases involving the treatment of an IPP. Regarding interrater reliability, the intraclass coefficient for the rating of item 2 (PUL) was 0.94 (95% CI: 0.89–0.97). The Fleiss Kappa for items 4 (presence of MLE) and 5 (presence of IPP) was 0.84 (95% CI: 0.66–1.02) and 0.85 (95% CI: 0.67–1.03), respectively. The agreement rate for items 6 (bladder neck and urethra morphology) and 7 (injection sequence) were 93% and 100%, respectively. Kendall’s W was 0.37 (*p* = 0.343) for item 8 (injection sites).

Evaluation of the operation videos showed that injections into the lateral lobes, both ventral and dorsal to the standard 3 and 9 o’clock positions in the lithotomy position, were performed in ten cases, with six cases involving dorsal injections and four involving ventral injections. PUL measurements were conducted in all cases, consistently demonstrating accuracy, with no instances of operators omitting or shortening the field of view. Injection boundaries, especially those near the bladder neck and verumontanum, were strictly respected in each case. In 78% of cases, injections were completed in one lobe before proceeding contralaterally, while in the remaining 22%, injections alternated between lobes. Vapor leakage was observed in three cases, and in one instance, an injection was repeated at the same site.

Upon reviewing the written operative notes, variability in the application of checklist items was observed. PVOL was documented in 17% of cases. Comments on the presence or absence of MLE and IPP were noted in 84% and 60%, respectively. The bladder neck-verumontanum distance was recorded in 81%, while the injection sequence was specified in only 8%. The total number of injections per lobe was documented in 96%; however, specific injection positions—including distance from the bladder neck, verumontanum, and spacing between injection points (critical for diagram plotting)—were detailed in only 30% of cases. Vapor leakage was reported in a single case, though it was observed in three cases upon video review.

## 4. Discussion

Rezum has demonstrated promising outcomes with low complication rates [5], even in high-risk patients like those under oral anticoagulants and platelet aggregation inhibitors [6]. Combined with the option to perform under local anesthesia or analgosedation, Rezum presents a choice for a wide spectrum of patients, from young seeking to preserve sexual function to polymorbid, catheter-dependent patients [6]. While this versatility makes it valuable for various indications, it presents challenges in comparing outcomes within highly diverse patient cohorts. Initial studies examining Rezum employed stringent inclusion criteria (e.g., urinary flow rate, prostate size) [5], and there is growing emphasis on the necessity for additional prospective real-world data with long follow-up [6,7]. Yet, even in rigorous efforts to gather such data, outcome interpretation could be complicated by intraoperative anatomical findings and surgical technique. Despite the prerequisite of a training course and a recommended surgical technique, Rezum remains a customizable procedure, given the technical specificity of surgeon-dependent choices regarding injection number and location [4].

Various injection strategies are investigated. Some advocate ‘less is more’ with a single injection per lobe to reduce complications and patient discomfort [7,8,9,10,11]. Others propose more injections [10], including a zig-zag approach with injections ventral and dorsal to 3 and 9 o’clock horizons, particularly for prostates larger than 80 mL. However, published data present contradictory findings regarding the impact of the number of injections on surgical retreatment rates [7,12]. Additionally, anecdotal consideration has been given to anterior injections. Despite developments in injection schemes and reported associations between injection number and adverse events [11], essential intraoperative details are often inaccessible, challenging to extract from operation notes, and infrequently reported in extensive literature series [5,13], complicating outcome comparison and underscoring the necessity of a standardized system for documenting operative technique.

SRPs and surgical checklists have demonstrated value across various fields. For example, the EAU strongly recommends using a bladder diagram for documenting cystoscopic findings in bladder cancer [14]. Using a surgical checklist at the time of transurethral resection can even improve risk group assignment and decrease bladder cancer recurrence rates [15]. Other examples of reporting systems that transformed urologic practice are the Prostate Imaging Reporting and Data System (PI-RADS) [16] and the Clavien-Dindo classification system, addressing the lack of consensus on defining and grading adverse postoperative events [17].

To our knowledge, this study introduces the first SRP for the Rezum procedure. Application of the Rezum-SRP revealed a wide range of injection densities and intervals, with lateral lobe injections placed ventrally and dorsally to the standard 3 and 9 o’clock positions, and an alternating sequence of injections between lobes. Differences in the mean number of injections per case were observed compared to other studies. Our study revealed a mean of 4.6 injections per case, aligning closely with McVary et al.’s [5] reported mean of 4.5 injections, but notably lower than Elterman et al.’s [13] mean of 11 injections per case. This discrepancy could potentially be attributed to variations in prostate size. Elterman et al. [13] reported a mean PVOL of 71.5 mL (ranging from 20 to 160 mL), which is more akin to our observed mean of 59.2 mL (ranging from 20 to 200 mL). Conversely, McVary et al.’s [5] study primarily included smaller prostates, with volumes <80 mL and a mean volume of 45.8 ± 12.9 mL. Despite these differences, the absence of data on injection density and interval at the patient level in these studies limits further analysis and a comparison of the study results. Insights into injection density were presented by Cindolo et al. [18], comparing patients meeting randomized clinical trial criteria with an unselected cohort undergoing Rezum. Despite statistically significant differences in PVOL (55 vs. 85 mL) and number of injections (7 vs. 9), no disparity was observed in injection density (10 mL PVOL per injection in both cohorts) [18]. PVOL and PUL appear to play a role in determining the necessary number of injections, but other patient-specific factors and treatment protocols may impact this relationship. Including details on injection density and intervals is recommended to improve outcome comparison, especially considering strategies advocating “less is more” approaches [7,8,9].

Apart from technical procedural nuances, further investigation is required into the interaction between anesthesia and treatment success, as anesthesia type could influence patient comfort and procedure efficacy. The time interval between local, intraprostatic anesthesia administration and the start of Rezum injections also warrants investigation due to potential interaction between the anesthetic and released steam. It is unclear whether intraprostatic anesthesia fluid affects tissue ablation extent by altering tissue properties or steam distribution. Integrating our SRP into operative reports could facilitate standardized documentation and enhance accessibility of critical information for analysis.

The steps noted in the operative report may mirror their importance to the surgeon and anticipating reporting particular steps in the documentation may improve the chances of achieving the desired outcome by framing the operator’s thinking. For instance, higher complication rates have been observed in cholecystectomies lacking a critical view of safety description in the operative note [19]. Similarly, it is conceivable that a Rezum checklist, detailing safety margins to the bladder neck and verumontanum or a 45° injection angle for the MLE, could underscore the importance of these steps and this way mitigate irritative urinary symptoms, ejaculatory dysfunction, and rectal irritation, respectively. Despite the recommendation to administer all injections sequentially within one lobe before proceeding contralaterally, we observed cases where injections alternated between lobes. Investigating injection sequence and vapor leakage may clarify associations with findings such as unchanged post-treatment endoscopic appearance of treated areas or the observed partial tissue response with the formation of tissue caverns or membranes. Finally, identifying an obstructive bladder neck, MLE, or IPP and whether they were addressed, may help analyze treatment failure [20] and guide salvage therapy.

The discussed points highlight the importance of establishing a standardized Rezum documentation to collect data in a uniform manner, making it comparable. The expert panel was able to achieve content validity for the SRP. This is also reflected in the high inter-rater reliability when applying the SRP to the assessment of Rezum operation videos, with excellent reliability observed for item 2 (ICC > 0.9), almost perfect agreement observed for items 4 and 5 (Fleiss Kappa > 0.81), 100% agreement for item 7, and a moderate to strong agreement for item 8 (Kendall’s W 0.3–0.4).

It is important to note this study has limitations that must be addressed. Interrater reliability was not assessed for Rezum cases involving checklist item 9 (additional procedures and adverse events), nor for checklist items 1 (prostate volume) and 10 (sign out), as these two aspects could not be adequately evaluated from surgical videos. Furthermore, interrater reliability for item 6 (bladder neck and urethral morphology) may have been insufficiently analyzed in this population, as most patients underwent urethrocystoscopy prior to treatment. This may have led to an underrepresentation of unexpected anatomical abnormalities (e.g., urethral strictures or bladder neck fibrosis).

Additionally, checklist items were rated by independent assessors who were not the operating surgeons, which could have introduced perceptual differences. This issue is relevant for the assessment of distances within the prostatic urethra, where inherent interobserver variation is expected. Nevertheless, no other measurement system exists, this rating is inherently operator-dependent, and this approach represented the only feasible method to assess interobserver variability within the same Rezum case. Reviewing the videos also allowed the assessors to verify whether certain procedural steps, such as safe distances to the bladder neck and verumontanum, were respected, whether non-standard injections were performed and whether events such as vapor-leakage occurred.

While the SRP enables precise documentation of injection sites, its complexity due to the high number of variables in the injection site matrix may complicate data interpretation. To mitigate this and ensure an accurate assessment of interrater reliability for this item, while accounting for minor variations in perceived distances, a weighted comparison against a reference standard was employed. Based on Kendall’s W interpretation for subjective ratings [21], which demonstrated a moderate to high agreement for item 8 (injection site ratings), it can be assumed that the diagrams will exhibit high user reliability when applied in clinical practice. Nonetheless, for future large datasets, a zonal separation of injection sites may be recommended to simplify data analysis. Such a zonal model can be developed once all possible injection positions have been systematically mapped, which is why a highly granular first iteration of the SRP was developed. Establishing the full spectrum of potential injection sites provides the foundational dataset required to understand which variations are relevant. Although it may be that small longitudinal differences along the prostatic urethra do not meaningfully influence tissue response or clinical outcomes, this assumption should be demonstrated rather than presupposed. A comprehensive map of all theoretical injection coordinates was therefore developed in this first version of the SRP. After documenting the entire range of possibilities, further simplification can be undertaken, such as consolidating individual coordinates into broader anatomical zones or sectors. This stepwise approach, providing users the possibility to document the procedure with maximal detail and progressing to evidence-based simplification, represents a necessary methodological progression toward a mature, optimized, and user-friendly SRP.

While the current analog version of the VAPOR-SRP is suitable for immediate scientific application and research purposes, a more user-friendly digital version is already under development. This digital version may be implemented as a dedicated electronic application or software module to facilitate integration into clinical routine. This refined version will integrate the 10-item surgical checklist and diagram options in an optimized layout, aiming to improve usability in routine clinical practice, provide an intuitive workflow that is also suitable for non-specialized or lower-volume centers, and enable the automated generation of operative reports.

The scope of the current work was the development and introduction of the SRP, the evaluation of its usefulness in assessing operative technique and the comparison to non-standardized documentation of Rezum procedures. The present study did not evaluate whether more complete or standardized SRP-based documentation translates into improved clinical outcomes (e.g., symptom improvement, quality of life, retreatment rates, or complication profiles). Consequently, the clinical value of the SRP remains preliminary and requires validation through outcome-linked analyses. A prospective, external, multi-institutional validation with large patient numbers in a real-word cohort considering variability in ratings due to case heterogeneity, and further matching to prospective outcome metrics is required to establish the SRP. Such efforts are planned as part of the SteamOne Study, a multicenter prospective database for Rezum (ClinicalTrials.gov ID: NCT05495633).

## 5. Conclusions

In summary, variability in the number and localization of injections relative to PVOL and PUL, along with missing critical information in non-SRP-standardized operative reports, has been observed. This highlights the need for standardized documentation, particularly regarding injection density and intervals, to facilitate data comparisons. Our proposed SRP, featuring an injection diagram and a surgical checklist, promises to improve the uniformity, completeness, and quality of documentation. This is essential for the standardized evaluation and comparison of Rezum outcomes and for identifying risk factors for treatment failures, from which future recommendations could be derived.

## Data Availability

The data presented in this study are available on reasonable request from the corresponding author due to privacy and confidentiality reasons.

## Supplementary Materials

The following supporting information can be downloaded at: https://www.mdpi.com/article/10.3390/jcm14238431/s1, Figure S1a–c: VAPOR-SRP diagrams for documentation of injection sites for PULs up to 10 cm; Figure S2: VAPOR-SRP diagram for documentation of injection sites in case of IPP treatment.

## Abbreviations

The following abbreviations are used in this manuscript:

AE	Adverse event
BPO	Benign prostatic obstruction
DVIU	Direct vision internal urethrotomy
EAU	European Association of Urology
ICC	Intraclass correlation coefficient
IPP	Intravesical prostatic protrusion
IQR	Interquartile range
LUTS	Lower urinary tract symptoms
MLE	Median lobe enlargement
MRI	Magnetic resonance imaging
PUL	Prostatic urethral length
PVOL	Prostate volume
SRP	Structured Reporting Protocol
TAUS	Transabdominal ultrasound
TRUS	Transrectal ultrasound
TUR	Transurethral resection
US	Ultrasound

## References

[1] McVary K.T., Gange S.N., Gittelman M.C., Goldberg K.A., Patel K., Shore N.D., Levin R.M., Rousseau M., Beahrs J.R., Kaminetsky J. (2016). Erectile and ejaculatory function preserved with convective water vapor energy treatment of lower urinary tract symptoms secondary to benign prostatic hyperplasia: Randomized controlled study. J. Sex. Med..

[2] Doppalapudi S.K., Gupta N. (2021). What is new with Rezūm water vapor thermal therapy for LUTS/BPH?. Curr. Urol. Rep..

[3] European Association of Urology (2024). Management of non-neurogenic male LUTS. EAU Guidelines, Presented at the EAU Annual Congress, Paris, France, 5–8 April 2024.

[4] Cantrill C.H., Zorn K.C., Elterman D.S., Gonzalez R.R. (2019). The Rezūm system: A minimally invasive water vapor thermal therapy for obstructive benign prostatic hyperplasia. Can. J. Urol..

[5] McVary K.T., Gange S.N., Gittelman M.C., Goldberg K.A., Patel K., Shore N.D., Levin R.M., Rousseau M., Beahrs J.R., Kaminetsky J. (2016). Minimally invasive prostate convective water vapor energy ablation: A multicenter, randomized, controlled study for the treatment of lower urinary tract symptoms secondary to benign prostatic hyperplasia. J. Urol..

[6] Bausch K., Zahiti L., Schrutt M., Kretschmer A., Wetterauer C., Halbeisen F.S., Ebbing J., Seifert H.H. (2023). Water vapor thermal therapy of lower urinary tract symptoms due to benign prostatic obstruction: Efficacy and safety analysis of a real-world cohort of 211 patients. World J. Urol..

[7] Babar M., Azhar U., Loloi J., Sayed R., Labagnara K., Zhu M., Tang K., Salami A., Singh S., Ines M. (2023). Water vapour thermal therapy (Rezum) outcomes at 4 years in relationship to the number of injections: Is the “less is more” treatment approach durable?. BJU Int..

[8] Hindley R. (2023). Is less indeed more for Rezum water vapour treatment of the lateral prostate lobes?. BJU Int..

[9] Aladesuru O., Amankwah K., Elterman D., Zorn K.C., Bhojani N., Te A., Chughtai B. (2022). Pilot study of “less is more” Rezum for treatment of BPH. Urology.

[10] Garden E.B., Shukla D., Ravivarapu K.T., Kaplan S.A., Reddy A.K., Small A.C., Palese M.A. (2021). Rezum therapy for patients with large prostates (≥80 g): Initial clinical experience and postoperative outcomes. World J. Urol..

[11] Babar M., Loloi J., Azhar U., Tang K., Ines M., Singh S., Iqbal N., Ciatto M. (2023). Rezum outcomes in relationship to number of injections: Is less more?. J. Endourol..

[12] Nguyen V., Leach M.C., Cerrato C., Nguyen M.V., Bechis S.K. (2024). Retreatment for lower urinary tract symptoms after water vapor thermal therapy. Urology.

[13] Elterman D., Bhojani N., Vannabouathong C., Chughtai B., Zorn K.C. (2022). Large, multi-center, prospective registry of Rezūm water vapor therapy for benign prostatic hyperplasia. Urology.

[14] European Association of Urology (2025). Non-muscle-invasive bladder cancer. EAU Guidelines, Presented at the EAU Annual Congress, Madrid, Spain, 21–24 March 2025.

[15] Suarez-Ibarrola R., Soria F., Abufaraj M., Dahlem R., Gontero P., Shariat S.F., Gontero P. (2019). Surgical checklist impact on recurrence-free survival of patients with non-muscle-invasive bladder cancer undergoing transurethral resection of bladder tumour. BJU Int..

[16] Israël B., Leest M.V., Sedelaar M., Padhani A.R., Zámecnik P., Barentsz J.O. (2020). Multiparametric magnetic resonance imaging for the detection of clinically significant prostate cancer: What urologists need to know. Part 2: Interpretation. Eur. Urol..

[17] Clavien P.A., Barkun J., de Oliveira M.L., Vauthey J.N., Dindo D., Schulick R.D., de Santibañes E., Pekolj J., Slankamenac K., Bassi C. (2009). The Clavien-Dindo classification of surgical complications: Five-year experience. Ann. Surg..

[18] Cindolo L., Campobasso D., Conti E., Uricchio F., Franzoso F., Maruzzi D., Viola L., Varvello F., Balsamo R., Ferrari G. (2023). Do patients treated with water vapor therapy and meeting randomized clinical trial criteria have better urinary and sexual outcomes than an unselected cohort?. J. Endourol..

[19] Stewart L., Hunter J.G., Wetter A., Chin B., Way L.W. (2010). Operative reports: Form and function. Arch. Surg..

[20] Whiting D., Noureldin M., Abdelmotagly Y., Johnston M.J., Brittain J., Rajkumar G., Emara A., Hindley R. (2022). Real-world early outcomes and retreatment rates following water vapour ablative therapy for symptomatic benign prostatic hyperplasia. Eur. Urol. Open Sci..

[21] Schmidt R.C. (1997). Managing Delphi surveys using nonparametric statistical techniques. Decis. Sci..

