# Supplementary material for: Introduction of a Structured Reporting Protocol and Surgical Checklist for Rezum Water Vapor Therapy (VAPOR-SRP)
Source: J Clin Med. 2025 Nov 27;14(23):8431. doi: 10.3390/jcm14238431 (PMC12692852; doi:10.3390/jcm14238431)
Supplement: Supplementary file 1 [file jcm-14-08431-s001.zip › Figure S1a.pdf]

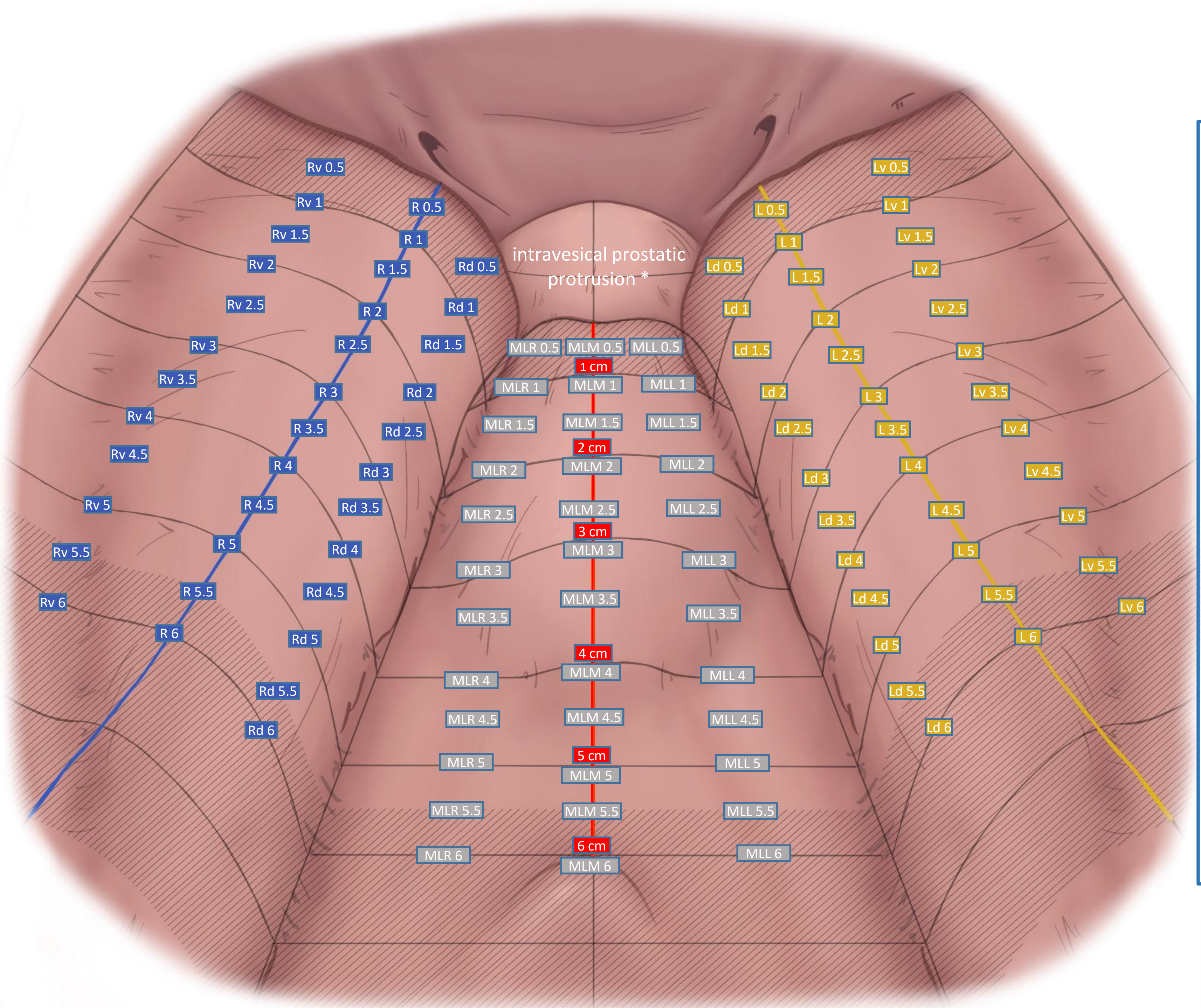

Bladder Neck-Verumontanum Distance: 4.5-6 cm

- Injection site axis
  - Blue line: 90° right horizon (9 o'clock in lithotomy position)
  - Yellow line: 90° left horizon (3 o'clock in lithotomy position)
  - Red line: longitudinal midline, bladder neck – verumontanum distance (prostatic urethral length)
- Striated zones: safety zones
- Distances:
  - Vertical distance between transverse lines: 1cm
  - Longitudinal distance between injection-site options: 0.5cm
- Abbreviations for injection-site options (the choice of injection site should be the one closest corresponding to the needle insertion point):
  - R: right, along the blue line
  - Rv: right, ventral (above the blue line)
  - Rd: right, dorsal (below the blue line)
  - L: left, along the yellow line
  - Lv: left, ventral (above the yellow line)
  - Ld: left, dorsal (below the yellow line)

  - MLR: median lobe enlargement right (right lateral to midline)
  - MLM: median lobe enlargement midline
  - MLL: median lobe enlargement left (left lateral to midline)
    - Additional options for median lobe enlargement injections:
      - Injection at a 45° angle
      - Perpendicular injection at a 90° angle
- Numbers next to abbreviations for injection-site options:
  - Distance from bladder neck in cm (4.5 – 6 cm)

* If an intravesical prostatic protrusion was treated, please see separate diagram
† If the bladder neck – verumontanum distance is > 4cm, please see appropriate diagram

All rights reserved by the authors.
