# Supplementary material for: Introduction of a Structured Reporting Protocol and Surgical Checklist for Rezum Water Vapor Therapy (VAPOR-SRP)
Source: J Clin Med. 2025 Nov 27;14(23):8431. doi: 10.3390/jcm14238431 (PMC12692852; doi:10.3390/jcm14238431)
Supplement: Supplementary file 1 [file jcm-14-08431-s001.zip › Figure S2.pdf]

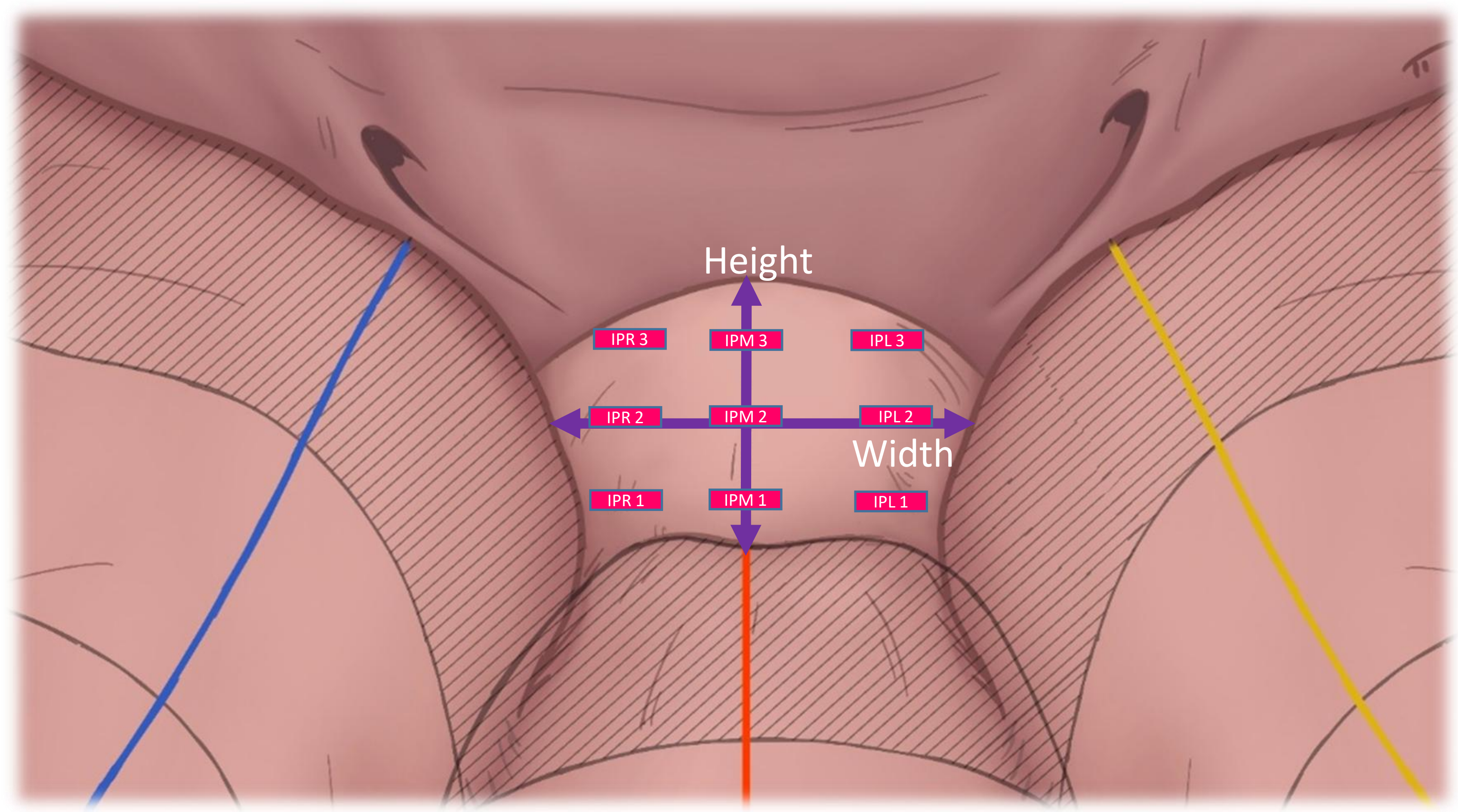

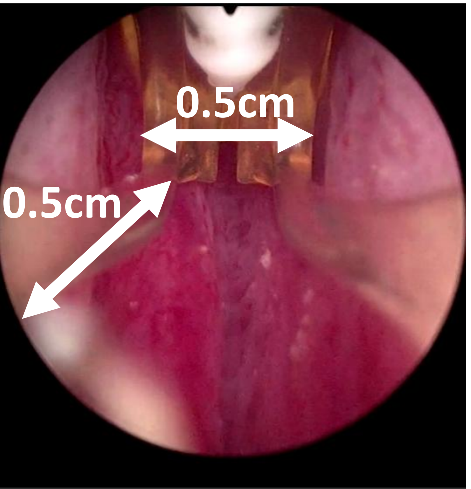

Intravesical Prostatic Protrusion

- Injection site axis
  - Blue line: 90° right horizon (9 o'clock in lithotomy position)
  - Yellow line: 90° left horizon (3 o'clock in lithotomy position)
  - Red line: longitudinal midline, bladder neck – verumontanum distance (prostatic urethral length)
- Striated zones: safety zones
- Height: distance from the dome of the intravesical prostatic protrusion to the bladder neck in cm
- Width: distance in cm measured at half the height, starting from the contact point of the IPP with the bladder neck or the lateral prostate lobe
- The intravesical prostatic protrusion is to be divided into 9 equal area zones, regardless of measurements. The choice of injection site should be the one closest corresponding to the needle insertion point.
- Abbreviations for injection-site options:
  - IPR: Intravesical prostatic protrusion right (1-3 equal area zones)
  - IPM: Intravesical prostatic protrusion middle (1-3 equal area zones)
  - IPL: Intravesical prostatic protrusion left (1-3 equal area zones)
    - Additional options for intravesical prostatic protrusion injections:
      - Injection at a 45° angle
        - Medial to lateral
        - Lateral to medial
      - Perpendicular injection at a 90° angle
- Picture at the bottom left: depiction of distances on the delivery device

All rights reserved by the authors.
